# Clinical and Electrophysiological Hints to TMS in De Novo Patients with Parkinson’s Disease and Progressive Supranuclear Palsy

**DOI:** 10.3390/jpm10040274

**Published:** 2020-12-12

**Authors:** Francesco Fisicaro, Giuseppe Lanza, Mariagiovanna Cantone, Raffaele Ferri, Giovanni Pennisi, Alessandra Nicoletti, Mario Zappia, Rita Bella, Manuela Pennisi

**Affiliations:** 1 Department of Biomedical and Biotechnological Sciences, University of Catania, Via Santa Sofia, 97-95123 Catania, Italy; drfrancescofisicaro@gmail.com (F.F.); manuela.pennisi@unict.it (M.P.); 2 Department of Surgery and Medical-Surgical Specialties, University of Catania, Via Santa Sofia, 78-95123 Catania, Italy; pennigi@unict.it; 3 Department of Neurology IC, Oasi Research Institute-IRCCS, Via Conte Ruggero, 73-94018 Troina, Italy; rferri@oasi.en.it; 4 Department of Neurology, Sant’Elia Hospital, ASP Caltanissetta, Via Luigi Russo, 6-93100 Caltanissetta, Italy; m.cantone@asp.cl.it; 5 Department of Medical and Surgical Sciences and Advanced Technologies, University of Catania, Via Santa Sofia, 87-95123 Catania, Italy; anicolet@unict.it (A.N.); m.zappia@unict.it (M.Z.); rbella@unict.it (R.B.)

**Keywords:** transcranial magnetic stimulation, cortical excitability, electrophysiology, neurodegeneration, parkinsonian syndrome, atypical parkinsonism

## Abstract

Background: Transcranial magnetic stimulation (TMS) can non-invasively probe cortical excitability in movement disorders, although clinical significance is still controversial, especially at early stages. We compare single-pulse TMS in two prototypic synucleinopathy and tauopathy—i.e., Parkinson’s disease (PD) and Progressive Supranuclear Palsy (PSP), respectively—to find neurophysiological differences and identify early measures associated with cognitive impairment. Methods: 28 PD and 23 PSP de novo patients were age-matched with 28 healthy controls, all right-handed and drug-free. Amplitude and latency of motor evoked potentials (MEP), central motor conduction time, resting motor threshold (rMT), and cortical silent period (CSP) were recorded through a figure-of-eight coil from the First Dorsal Interosseous muscle (FDI), bilaterally. Results: Mini Mental Examination and Frontal Assessment Battery (FAB) scored worse in PSP; PD had worse FAB than controls. Higher MEP amplitude from right FDI in PD and PSP than controls was found, without difference between them. CSP was bilaterally longer in patients than controls, but similar between patient groups. A positive correlation between FAB and rMT was observed in PSP, bilaterally. Conclusions: Despite the small sample size, PD and PSP might share, at early stage, a similar global electrocortical asset. rMT might detect and possibly predict cognitive deterioration in PSP.

## 1. Introduction

Among movement disorders, an accurate and early diagnosis of the most common tauopathy—i.e., Progressive Supranuclear Palsy (PSP)—and the differential diagnosis from the prototypic α-synucleinopathy—i.e., Parkinson’s disease (PD)—is often challenging, particularly at their early stages [1,2,3]. In addition to the typical histopathological lesion observed in PD (i.e., degeneration of the substantia nigra pars compacta), the thinning of the medial frontal (premotor and supplementary motor) and posterior cingulate cortex has been described [4]. Furthermore, dopamine depletion can induce a functional reorganization of the motor maps [5]. In PSP, argyrophilic and tau-positive tufted astrocytes, neurofibrillary tangles, coiled bodies, and thread-like processes are found not only in the basal ganglia and brainstem nuclei, but also in the primary motor cortex (M1) [6]. The different histological substrates of these diseases might be associated with specific neurophysiological characteristics. In this context, the role of non-invasive brain stimulation techniques, such as transcranial magnetic stimulation (TMS), may help in disentangling this complex pathophysiology.

TMS is a non-invasive electrophysiological technique able to assess the intracortical excitability and the cortico-spinal conductivity in vivo and in “real time”, thus being used in clinical practice [7,8,9,10,11], research settings [12,13,14,15], and experimental treatments [16,17]. Single-pulse stimulation is routinely used to assess basic features of motor evoked potentials (MEPs), including MEP latency and amplitude, central motor conduction time (CMCT), and some global measures of excitability, such as the resting motor threshold (rMT) and the cortical silent period (CSP). rMT and MEP reflect not only the conductivity of the cortico-spinal tract, but also the excitability of the M1 and nerve roots, as well as the conduction along the peripheral motor pathway till the muscles [8]. While CSP is believed to be due to inhibitory mechanisms at the M1 level, spinal components (such as the Renshaw inhibition) are thought to contribute, although only to the first 50–60 ms of this suppression [18].

Since the early studies [19], indeed, TMS has been used to explore the inhibitory and excitatory interactions of both motor and non-motor cortical regions, within and across cerebral hemispheres, thus providing insights into the intracortical and intercortical mechanisms underlying the role of different brain regions in cognitive processes, motor control, and plastic changes after a brain lesion or during the course of a neurodegenerative process [20]. Used in combination with neuroimaging, TMS also provides information on the functional connectivity between different motor and non-motor regions and on the relationship between pathophysiological processes and specific brain areas [20]. Although the abnormalities revealed by TMS are not disease-specific [18], there may be distinct neurophysiological changes that co-segregate in each dementing illness, consistent with the involvement of specific neurobiological substrates [19,21,22,23,24]. These indexes have also been proposed for the early detection of cognitive impairment, the monitoring of disease progression, and the evaluation of treatment response [25,26].

A new concept of the M1 has also emerged, in which motor cortical output is influenced also by non-primary motor areas, including ventral and dorsal premotor cortex, and supplementary motor area, and even by non-motor regions (such as the cingulate cortex) [20]. Accordingly, although not always clinically evident, the involvement of motor areas in dementia has been demonstrated, with changes in motor areas that may be secondary to direct structural alterations caused by the disease process or, more often, to indirect plastic remodeling mechanisms [19]. Finally, by evaluating the effects of agonists or antagonists for specific neurotransmitters, TMS can selectively and non-invasively probe the functioning of glutamatergic, gamma-aminobutyric acid (GABA)-ergic and cholinergic cortical circuits [27], which are all implicated in cognitive and movement disorders.

When applied to movement disorders, TMS can characterize the cortical excitability at the final motor output stage and its modulation by altered basal ganglia activity [28]. Histology [29] and functional imaging in vivo studies [30] demonstrated that the direct dopaminergic innervation of M1 degenerates relatively early in PD patients, a finding which is in line with a local reduction in its metabolism [31]. Furthermore, electrophysiological effects of dopaminergic degeneration also on subcortical motor structures are well documented, as well as the evidence that M1 activity is influenced by existing PD therapies, including not only l-3,4-dihydroxyphenylalanine (l-DOPA) but also deep brain stimulation of the subthalamic nucleus, as very recently reviewed [32].

Overall, in PD patients, the majority of TMS studies suggests an imbalance of M1 excitability towards a state of reduced inhibition. Specifically, a marked reduction in the short-interval intracortical inhibition (SICI), which is a measure of the GABA-A receptor transmission, has been reported, suggesting an impaired intracortical inhibitory activity which partially reverted after dopaminergic therapy [33,34,35,36]. Shortening of CSP duration has been reported in PD patients, particularly in the early untreated stages, and correlated with limb rigidity [37]. Regarding the short-latency afferent inhibition (SAI), which is thought to reflect the central cholinergic functioning, evidence is conflicting, with researchers reporting reduced [38,39], normal [40], or increased values [41]. Finally, most studies have reported that rMT is normal in PD [34,36,42], whereas increased MEP amplitude at rest has been found [43,44].

In PSP, earlier studies revealed CMCT abnormality, although only in a subgroup of patients with a long disease duration [37,45,46]. Reduced SICI, changes in CSP duration, and increased MEP amplitude have been reported and appeared to correlate with the disease progression [46,47,48]. More recent work also showed changes in cortical excitability and synaptic plasticity [47,49,50,51]. Regarding CSP, a longer duration has been reported in PSP, whereas the opposite has been observed in PD patients [52]. However, CSP is highly variable depending on several factors (e.g., TMS intensity, selection of target muscle, coil shape, and position) [53], and its significance in clinical practice is still controversial.

As TMS can detect early electrophysiological changes of movement disorders, we examined and compared single-pulse TMS in de novo patients with PD and PSP with a relatively short disease duration. As mentioned, indeed, studies directly comparing PD and PSP at their early stages are scarce and rather conflicting, whereas those correlating clinical-cognitive data with TMS findings in early non-demented patients are lacking. We hypothesized that the two groups of patients may show different neurophysiological patterns and that specific TMS measures might be associated with cognitive impairment, which is known to be more prominent in PSP than in PD.

## 2. Materials and Methods

### 2.1. Subjects and Assessment

We consecutively recruited 28 de novo patients with idiopathic PD (median age 63.5 years, range 59.0–69.5; 18 males) and 23 de novo patients with PSP (median age 67 years, range 63.0–72.0; 11 males) from the Neurology Department of the “Azienda Ospedaliera Universitaria Policlinico Gaspare Rodolico-San Marco” of Catania (Italy), from November 2018 to September 2020. The control group consisted of 28 age-matched healthy subjects (median age 65 years, range 58.5–69.0; 16 males), recruited from the TMS Lab of the above-mentioned Institution. All participants were right-handed, as assessed by the Edinburgh Handedness Inventory [54]. Controls were drug-free, did not have any history of neurological or psychiatric disorders, and their neurological examination and brain magnetic resonance imaging (MRI) were both normal.

All patients had a diagnosis of probable PD based on the clinical diagnostic criteria of the Movement Disorder Society [55], or PSP following the criteria of the National Institute of Neurological Disorders and Stroke—Society for PSP [56]. The median disease duration was 2 years (range 2–4) in the PD group and 3 years (range 2–4) in the PSP group. DaTscan single-photon emission computed tomography and magnetic resonance parkinsonism index, performed in all patients, were compatible with an early stage of the diseases [57,58]. Among PD patients, 22 had an akinetic-rigid form, whereas the other 6 exhibited a mixed presentation, with a predominant akinetic-rigid phenotype; brain MRI was normal in all of them. The majority of PD patients (25 out of 28) presented an asymmetry of their motor manifestations, being the right side more clinically affected than the left side; the remaining three exhibited a bilateral presentation. All PSP patients had a bilateral akinetic-rigid syndrome with predominant symptoms in the right limbs, which poorly responded to l-DOPA; brain MRI showed varying degrees of brainstem, basal ganglia, and frontal lobe atrophy. All patients were clinically evaluated by using the Unified Parkinson’s Disease Rating Scale (UPDRS)—part III (motor examination) [59] and the Hoehn and Yahr (H-Y) scale [60].

None of the participants had major neurocognitive disorder (dementia) on the basis of the latest diagnostic criteria of the Diagnostic and Statistical Manual of Mental Disorders, Fifth Edition [61]. The Mini Mental State Examination (MMSE) [62] was administered to all subjects in order to screen cognitive functioning, as well as the Frontal Assessment Battery (FAB) [63] to assess the frontal lobe abilities, and the Hamilton Depression Rating Scale [64] to quantify any symptom of depression. Both MMSE and FAB scores were adjusted for age and educational level for each subject. Clinical and neuropsychological variables were evaluated independently by different investigators (AN and RB), and the TMS operators (GL and MP) were blinded to the clinical scores. Patients were drug-naïve and no other medication able to affect cortical excitability was assumed before TMS [27,65,66].

Exclusion criteria were: other neurological diseases (e.g., traumatic head or back injury, stroke or chronic cerebrovascular disease, spinal cord injury or any other spinal cord disease, any other movement disorder, any inflammatory or demyelinating disease, tumors, etc.); peripheral neuropathies, radiculopathies, or neuromuscular disorders; previous cranial or spinal surgery; major psychiatric diseases; acute, advanced, or chronic not compensated medical illnesses (including diabetes, hypothyroidism, and neoplasm); history or presence of seizures, implanted biomedical devices (i.e., pacemaker, prosthesis, intracranial clips), pregnancy at the time of testing, or any other contraindication to TMS [7]. Although evidence of cervical disc protrusions was present in some patients, none had any clinical or radiological sign of cervical cord or spinal root compression. A conventional electroencephalography was preliminary performed prior to TMS to exclude predisposition to seizures.

The study was carried out by trained operators in accordance with the latest recommendations of the International Federation of Clinical Neurophysiology (IFCN) for the diagnostic use of TMS [8]. All the experimental procedures were approved by the Ethics Committee of the “Azienda Ospedaliera Universitaria Policlinico Gaspare Rodolico-San Marco” (approval number: 9/2018/PO) and carried out following the rules of the Declaration of Helsinki of 1975, revised in 2013. All subjects gave their signed informed consent for inclusion before they participated in the study.

### 2.2. Transcranial Magnetic Stimulation

TMS was performed using a high-power Magstim 220 mono-pulse magnetic stimulator (Magstim Co., Whitland, Dyfed, UK). A figure-of-eight coil (external loop diameter 90 mm) was held tangentially over the M1 of each hemisphere, at the optimum scalp position (“hot spot”) to elicit MEPs in the contralateral First Dorsal Interosseous (FDI) muscle of each hand, with the induced current flowing in a posterior–anterior direction, as recommended [8]. Once located, the hot spot was marked on the scalp with a soft-tip pen.

Electromyographic (EMG) activity was recorded with silver/silver-chloride disposable self-adhesive and self-conductive surface electrodes. The active electrode was placed over the muscular belly of the target muscle, the reference was positioned distally at the metacarpal-phalangeal joint of the index finger, and the ground was on the dorsal face of the wrist. MEPs were amplified and filtered (bandwidth 3–3000 Hz), and recorded by using a 2-channel Medelec Synergy system (Oxford Instruments Medical, Inc, Surrey, UK).

The rMT was defined as the lowest stimulus intensity able to elicit MEP at rest of an amplitude >50 μV in at least 5 of 10 trials, according to the above-mentioned guidelines [8]. Five reproducible MEPs during moderate active muscle contraction (about 10–20% of the subject’s maximum voluntary contraction, by using a strain gauge) were elicited. Among them, the MEP with the shortest latency was considered for CMCT calculation, according to the IFCN guidelines [8]. CMCT was calculated by subtracting the conduction time in peripheral nerves obtained by magnetic stimulation of the cervical root, from the shortest MEP cortical latency obtained with a stimulus intensity set at 130% of the rMT. To ensure reproducibility, three peripheral motor responses were recorded at rest and averaged. Peak-to-peak MEP amplitude during active contraction level was also calculated.

The CSP was determined with an approximately 50% of maximum tonic voluntary contraction of the FDI muscle, induced by single TMS pulses delivered at 130% of rMT. As recommended [8], the mean CSP duration of 7 rectified trials was calculated. Namely, in a single trial, the CSP was measured as the time elapsing from the onset of the MEP until the recurrence of voluntary tonic EMG activity.

Since PD and, to a lesser extent, PSP are characterized by an asymmetry of the motor manifestations, side-to-side difference was also considered for each TMS index, with “right” and “left” referred to the recording side of the target muscle. To avoid motor fatigue and inter-trial variability, a pause of 20 s was taken after every stimulus. A continuous EMG audio–visual feedback at high gain assisted the participants in maintaining a complete muscle relaxation. Trials containing any type of artifact were removed. Similarly, we excluded trials contaminated by EMG activity at rest (indicating a non-relaxed muscle), as well as those “active” trials (during contraction) with excessive EMG voluntary activity that made a reliable recognition of the onset of MEP cortical latency difficult or doubtful.

In sum, the following TMS measurements and repetitions were obtained for each participant:

- rMT (%): at least 5 of 10 trials with a MEP amplitude >50 μV at rest;
- MEP latency (ms): 5 reproducible recordings during active contraction;
- MEP amplitude (mV): 5 reproducible recordings during active contraction;
- peripheral motor latency (ms): 3 reproducible recordings at rest;
- CSP (ms): 7 reproducible rectified trials during active contraction.

All measurements were conducted while subjects were seated in a comfortable armchair, with their arms maintained relaxed in the same position throughout the procedure. All recordings were performed in the same laboratory, equipment, and experimental conditions, by the same operators and at the same time of the day (09:30–11:30 a.m.), in order to exclude possible confounding due to the circadian rhythm. All data were collected on a dedicated PC and stored with an ad hoc software for off-line analysis [67].

### 2.3. Statistical Analysis

Because of the non-normal distribution of some variables, the differences between the continuous variables obtained in the different groups of subjects (patients and controls) were evaluated by means of the non-parametric Kruskal–Wallis ANOVA, followed by the Mann–Whitney test for independent datasets, used as a post hoc test for the comparison of each pair of groups, when appropriate. Within-group comparisons were carried out by means of the Wilcoxon test, and correlations were evaluated by means of the Spearman’s rho. *P* values were considered statistically significant when <0.05.

Because of the relatively low number of subjects and in order to limit the possibility to miss significant differences, we computed the effect size for all comparisons by calculating the Cohen’s *d* and checked for instances in which a “large” effect size was present, which is commonly accepted to be indicated by a Cohen’s *d* value >0.8 [68].

## 3. Results

All participants underwent TMS without any discomfort or undesired effect. The clinical and demographic features of participants are summarized in Table 1. As shown, the three groups were similar in terms of age, height, disease duration and severity, motor impairment, and mood status. MMSE and FAB scored worse in PSP patients than controls, but they did not significantly differ from the scores of PD subjects. PD patients exhibited a significantly lower FAB score than controls.

As shown in Table 2, single-pulse TMS revealed a significantly higher MEP amplitude from the right FDI muscle in both PD and PSP patients compared to controls, but not between the two groups of patients. Similarly, CSP duration was significantly and bilaterally longer in patients than controls, but it was similar between PD and PSP. No other difference was observed.

Table 3 shows the right-to-left differences within the patients’ groups. In the PSP group, MEP amplitude from the right FDI was significantly higher and MEP latency was significantly prolonged compared to the contralateral side, although these differences did not have a large effect size.

**Figure 1:**
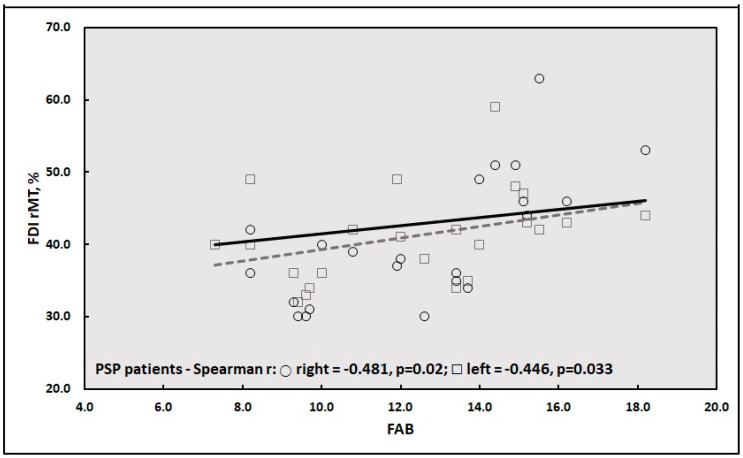
Correlation between FAB score and rMT from the left and right FDI muscles in PSP patients. Legend (in alphabetical order): FAB: Frontal Assessment Battery; FDI: First Dorsal Interosseous; PSP: Progressive Supranuclear Palsy; rMT: resting motor threshold; lines: linear regression lines.

Finally, the correlation analysis between clinical-cognitive and TMS data in the three groups disclosed a significant positive correlation between FAB score and rMT from both hemispheres in PSP patients (Figure 1).

## 4. Discussion

### 4.1. Main Findings

Compared to the existing literature, we first documented a similar global electrocortical asset in PD and PSP patients at their early stage and proposed the potential role of the rMT in the early detection and possibly prediction of cognitive deterioration in PSP subjects. We also confirm that the excitability of specific cortical networks is abnormal in both PD and PSP patients, although they did not exhibit distinctive electrocortical patterns to single-pulse TMS. In this scenario, a previous study suggested that some global measures of motor cortex inhibition (i.e., CSP) differed between the two conditions, the CSP being longer in PSP and shorter in PD with respect to healthy controls [52]. However, our findings support the hypothesis that, at their early stages, a similar level of impairment of motor cortex inhibition might occur. Interestingly, patients’ MEP latency and CMCT were similar to those of healthy controls, suggesting a normal cortico-spinal conductivity. This finding might help the differential diagnosis with other atypical parkinsonisms (APs)—i.e., Multiple Systemic Atrophy, which typically exhibits prolonged CMCT [52].

A role was previously proposed for neurophysiological testing in the differential diagnosis between PD and APs and between the various APs [37,46,69]. However, owing to the relatively low sensitivity of the majority of findings and the lack of post mortem diagnostic confirmation in most of the studies, it is not yet possible to conclude that neurophysiological techniques can reliably differentiate between these disorders [52]. The inconsistencies may be in part due to the heterogeneity of the patients studied, the high inter-individual variability of parameters, the differences in disease severity, the pre-activation of target muscles, and the dopaminergic state. Conversely, some follow-up studies indicate that longitudinal assessments using neurophysiological techniques may provide useful surrogate biomarkers [52], although further evidences are needed. Based on the results of the present study, we would suggest that PD and PSP could not be early differentiated on the basis of single-pulse TMS alone and that more advanced protocols (e.g., paired-pulse TMS, SAI, paired-associative stimulation) should be considered.

The significant increase in MEP amplitude in both groups of patients compared to controls, along with the previous finding of abnormally enhanced MEP amplitude after theta-burst stimulation in PSP patients [47], may be interpreted as a status of M1 hyperexcitability, likely through the basal ganglia-motor cortex loop [51,70], similarly to that posited in PD patients [42,71]. A possible explanation is that the enhanced excitability may reflect an increased glutamatergic excitatory interneuronal activity. The excessive glutamate release, indeed, is a key feature in the excitotoxicity model in cultured cortical neurons, as reported in several neurodegenerative diseases [72,73,74]. Therefore, an imbalanced glutamatergic activity may take place in both synucleinopathy and tauopathy [47]. Translationally, the increased MEP amplitude in both PD and PSP might suggest that the motor output neurons and the cortico-spinal tract are not severely affected [46], as also confirmed by the lack of any clinical motor deficit in our patients. In this context, the fact that both groups of patients had higher MEP amplitude from the right FDI compared to controls can be ascribed to the right handedness of these subjects, as previously reported in normal subjects [75,76], and possibly to the clinical asymmetry of their motor manifestations.

The same factors might explain the higher MEP amplitude obtained from the right side compared to the contralateral side within patients’ groups, although with a statistically significant difference in the PSP group only. In this context, pioneer neuroanatomical studies showed that the dominant hemisphere may have higher corticospinal tract density [77,78], although the neurophysiology underlying brain asymmetries is still controversial, with some authors suggesting that the dominant hemisphere exhibits larger cortical representation areas with lower excitability [79], while others report the opposite [80,81], or even no difference [82,83,84]. It has been recently demonstrated that a laterality asymmetry possibly leads to a more pronounced MEP distribution on the dominant hemisphere compared to the non-dominant side in healthy right-handers [85], and that patients with motor and movement disorders exhibit disruptions of motor unit recruitment and discharge patterns [86,87,88,89], which may affect dominant and non-dominant sides differently [90]. However, the lack of similar data in PSP precludes a better understanding of laterality asymmetries in these patients and stimulates further studies on this intriguing topic.

However, these observations do not fully explain why a markedly prolonged CSP in both PD and PSP patients with respect to controls was also found. Regarding the inhibitory components of the cortical excitability, one should keep in mind that, although these measures are known to reflect the activity of a main transmission pathway, they are actually influenced by different neurotransmitters, since they are sensitive to the global weight of several neurochemical pathways and circuitries from both cortical and subcortical inputs [66]. Therefore, the significant prolongation of CSP observed in PD and PSP patients, but without differences between them, might suggest that unbalanced excitatory and inhibitory cortical activity in M1 would occur similarly in these patients. Accordingly, a reduced SICI has been reported in both PD [33,34,35,36] and PSP [47], adding further support the hypothesis of an unbalanced cortical facilitatory and inhibitory circuitry within the M1 of both disorders. Translationally, this would imply the involvement of both glutamatergic and GABAergic systems in the cortical pathology of PD and PSP.

Regarding cognition, although not demented, a lower MMSE was detected in PSP subjects and a lower FAB in both groups of patients compared to controls. This finding is in line with the evidence of a worse global cognitive status and executive performance in PSP than PD and other APs, thus supporting a neuropsychological profile at higher risk for progression into dementia in these patients [91]. Accordingly, a novel finding of our study is the significant correlation between FAB and rMT in PSP patients. In particular, with worse FAB scores, PSP patients tended to exhibit a reduced rMT bilaterally, thus suggesting a global higher excitability from both hemispheres. Given that a reduced rMT, although not disease-specific, is a finding stably observed in degenerative dementias [19] and that, unlike MMSE, FAB has been considered a reliable test to assess cognitive impairment in these patients [91], the correlation we found might possibly disclose early cognitive and TMS markers of cognitive deterioration in PSP.

On the other hand, earlier independent investigations have demonstrated that different neurological diseases may exhibit similar TMS profiles. For instance, among cognitive disorders, it has been shown that patients with Alzheimer’s disease and subcortical ischemic vascular dementia can share common TMS features [92]. This suggests the existence of mechanisms that partially overlap and probably act in the same neurophysiological way, although they are, at least in principle, different in both localization (cortical versus subcortical) and origin (degenerative versus vascular) [93]. This alteration might promote a functional neuroplastic rearrangement allowing the preservation of motor programming and execution despite disease progression [92,94,95]. Conversely, the lack of correlation between clinical and MEP features in both PD and PSP patients can be explained by considering that TMS basically explores the M1, whereas the UPDRS–part III and the H-Y scale evaluate several aspects of motor and functional status, respectively. Since there is as yet little evidence of the clinical-neurophysiological correlations in PD and APs [46,96], the present study provides further insights, although further research is warranted.

### 4.2. Limitations

Some limitations should be mentioned. First, as usual in TMS studies, the sample size was small, although the patients were carefully screened, thus making the samples very homogeneous. Accordingly, in order to examine comparable groups, patients were selected to be as homogeneous as possible in terms of clinical features, disease duration, and severity. Additionally, control subjects were matched for age, sex, and height to both groups of patients. Furthermore, in order to limit the possibility of missing significant differences, we included a careful evaluation of the effect size by calculating the Cohen’s *d*. Indeed, the statistical analysis indicated a significant difference in all instances in which a “large” effect size was present, which is commonly accepted to be indicated by a Cohen’s *d* value >0.8. Nevertheless, further stratifications could not be performed—e.g., based on clinical phenotype or on the clinically affected side. Therefore, since the low population size remains a major limitation of the study, this should be considered as a pilot study needing further validation.

Second, a more precise estimation of the MEP size was obtained through the amplitude ratio—i.e., the ratio between the maximal transcranially evoked MEP amplitude and the maximal distally evoked compound motor action potential. Similarly, CMCT was not calculated by eliciting the F-waves. Additionally, a peripheral nerve conduction velocity study was not performed, although all subjects recruited did not have any sign or history of peripheral nerve pathology.

Another caveat is that only de novo patients with non-severe clinical phenotypes were enrolled and, therefore, longitudinal clinical, cognitive, and TMS investigations are needed. As a consequence, the present results should be considered as descriptive and the monitoring with serial MEP recordings will clarify the role and the pathophysiological weight of our findings over time.

Given that the right side was more clinically affected, we cannot exclude that the evaluation of the rMT from the right FDI muscle might have been influenced by the difficulty to obtain a complete muscle relaxation. However, in addition to the continuous EMG audio-visual feedback at high gain, we checked continuously that subjects remained relaxed as much as possible during the exam. Moreover, as stated, all the trials containing any type of artifact, as well as those trials contaminated by EMG activity at rest, were removed.

Finally, this study used basic MEP features, which represent a routine diagnostic application of TMS. As known, TMS can evaluate different parameters, such as the paired-pulse-derived measures, SAI, and indexes of interhemispheric functioning, although that was beyond the primary target of this clinically-oriented study. Overall, we acknowledge that errors in the study design might still have been present, although all possible measures to prevent them have been adopted.

## 5. Conclusions

Notwithstanding the limitations of the study (especially the small sample size), PD and PSP might share, at their early stage, a similar global electrocortical asset. Specific TMS metrics may be useful for monitoring PSP subjects at risk for cognitive deterioration. Advanced TMS, together with clinical-cognitive and structural-functional neuroimaging data, may lead to the detection of abnormalities more closely related to the specific pathological substrate of these diseases. Future translational applications will include the identification of markers of disease progression and response to pharmacological treatment in a wide range of movement disorders.

## Figures and Tables

**Table 1:** Clinical-demographic and neuropsychological features of the three groups.

Variable	Group 1 (Controls; *n* = 28)			Group 2 (PD; *n* = 28)			Group 3 (PSP; *n* = 23)			Kruskal–Wallis ANOVA			*post hoc*		
	Median	Lower	Upper	Median	Lower	Upper	Median	Lower	Upper	H_(2,79)_	*p*	Effect Size Cohen’s *d*	1 vs. 2	1 vs. 3	2 vs. 3
													*p*	*p*	*p*
Age, years	65.0	58.5	69.0	63.5	59.0	69.5	67.0	63.0	72.0	3.113	NS	0.244	-	-	-
Height, cm	162.0	160.0	170.0	163.5	160.0	170.0	163.0	155.0	170.0	0.105	NS	-	-	-	-
Disease duration, years	-	-	-	2.0	2.0	4.0	3.0	2.0	4.0	-	-	-	-	-	NS
Hoehn-Yahr	-	-	-	2.0	2.0	2.5	2.5	2.0	3.0	-	-	-	-	-	NS
UPDRS-ME	-	-	-	31.0	25.5	38.5	32.0	25.0	45.0	-	-	-	-	-	NS
MMSE	28.6	27.0	30.0	27.5	25.9	28.9	27.2	25.4	28.3	6.263	**0.044**	0.488	NS	**0.022**	NS
FAB	16.9	14.3	18.0	15.0	11.4	16.4	12.6	9.6	14.9	18.657	**0.0001**	**1.060**	**0.04**	**0.00005**	NS
HDRS	4.0	2.0	6.5	5.0	3.0	8.5	5.0	2.0	7.0	3.409	NS	0.275	-	-	-

Legend (in alphabetical order): FAB: Frontal Assessment Battery; HDRS: Hamilton Depression Rating Scale; MMSE: Mini Mental State Examination; NS: not significant; PD: Parkinson’s disease; PSP: Progressive Supranuclear Palsy; UPDRS-ME: Unified Parkinson’s Disease Rating Scale–part III (motor examination); numbers in bold: statistically significant *p* values and large (>0.8) effect sizes.

**Table 2:** Single-pulse transcranial magnetic stimulation (TMS) data obtained from all participants.

Variable	Group 1 (Controls; *n* = 28)			Group 2 (PD; *n* = 28)			Group 3 (PSP; *n* = 23)			Kruskal–Wallis ANOVA			*post hoc*		
	Median	Lower	Upper	Median	Lower	Upper	Median	Lower	Upper	H_(2,37)_	*p*	Effect Size Cohen’s *d*	1 vs. 2	1 vs. 3	2 vs. 3
													*p*	*p*	*p*
Right FDI MEP amplitude, mV	3.9	2.3	5.1	7.9	6.4	10.2	7.9	5.6	11.1	23.879	**0.00001**	**1.272**	**0.00001**	**0.00015**	NS
Right FDI MEP latency, ms	20.7	19.9	21.9	20.4	19.3	21.3	19.7	18.6	21.2	4.407	NS	0.362	-	-	-
Right FDI CMCT, ms	6.5	6.0	7.3	5.9	5.3	6.3	5.9	5.2	6.8	8.063	NS	0.589	-	-	-
Right FDI rMT, %	43.0	39.0	45.5	37.5	35.0	45.5	39.0	34.0	49.0	3.178	NS	0.251	-	-	-
Right FDI CSP, ms	69.4	58.5	87.2	125.4	95.0	135.2	131.8	92.8	162.0	23.131	**0.0001**	**1.241**	**0.00007**	**0.00004**	NS
Left FDI MEP amplitude, mV	4.6	4.0	6.0	6.1	4.2	8.2	6.4	4.0	9.2	3.234	NS	0.257	-	-	-
Left FDI MEP latency, ms	19.2	18.4	20.6	20.1	19.5	21.1	19.3	18.1	21.1	5.262	NS	0.424	-	-	-
Left FDI CMCT, ms	5.5	5.1	6.5	5.9	5.1	6.5	5.8	4.9	6.8	0.406	NS	-	-	-	-
Left FDI rMT, %	40.0	38.0	43.5	38.0	35.0	41.5	41.0	36.0	44.0	3.247	NS	0.258	-	-	-
Left FDI CSP, ms	74.5	60.5	117.0	131.5	79.3	139.0	146.3	101.0	151.0	12.807	**0.0017**	**0.814**	**0.035**	**0.00062**	NS

Legend (in alphabetical order): CMCT: central motor conduction time; CSP: cortical silent period; FDI: First Dorsal Interosseous muscle; K–W: Kruskal–Wallis; MEP: motor evoked potential; NS: not significant; PD: Parkinson’s disease; PSP: Progressive Supranuclear Palsy; rMT: resting motor threshold; TMS: transcranial magnetic stimulation; numbers in bold: statistically significant *p* value sand large (>0.8) effect sizes.

**Table 3:** Right-to-left difference of TMS parameters within the PD and PSP group.

Variable	PD Group				PSP Group			
	Wilcoxon’s Test			Effect Size Cohen’s *d*	Wilcoxon’s Test			Effect Size Cohen’s *d*
	Valid	Z	*p*		Valid	Z	*p*	
Right versus left FDI MEP amplitude	27	1.670	0.095	0.321	22	2.581	**0.001**	0.550
Right versus left FDI MEP latency	25	0.740	0.459	0.148	21	2.468	**0.0135**	0.539
Right versus left FDI CMCT	28	0.079	0.936	0.015	20	0.317	0.751	0.071
Right versus left FDI rMT	25	0.673	0.501	0.135	23	0.395	0.693	0.082
Right versus left FDI CSP	28	0.285	0.776	0.054	23	0.426	0.670	0.089

Legend (in alphabetical order): CMCT: central motor conduction time; CSP: cortical silent period; FDI: First Dorsal Interosseous muscle; MEP: motor evoked potential; PD: Parkinson’s disease; PSP: Progressive Supranuclear Palsy; rMT: resting motor threshold; TMS: transcranial magnetic stimulation; NS: not significant; numbers in bold: statistically significant *p* values.

## References

[1] Osaki Y., Ben-Shlomo Y., Lees A.J., Daniel S.E., Colosimo C., Wenning G., Quinn N. (2004). Accuracy of clinical diagnosis of progressive supranuclear palsy. Mov. Disord. Off. J. Mov. Disord. Soc..

[2] Lanza G., Papotto M., Pennisi G., Bella R., Ferri R. (2014). Epileptic seizure as a precipitating factor of vascular progressive supranuclear palsy: A case report. J. Stroke Cerebrovasc. Dis. Off. J. Natl. Stroke Assoc..

[3] Lanza G., Papotto M., Pennisi G., Bella R., Ferri R. (2015). Unusual presentation of atypical akinetic-rigid syndrome after liver transplantation: A case report and review of the literature. Acta Med. Mediterr..

[4] Zarei M., Ibarretxe-Bilbao N., Compta Y., Hough M., Junque C., Bargallo N., Tolosa E., Martí M.J. (2013). Cortical thinning is associated with disease stages and dementia in Parkinson’s disease. J. Neurol. Neurosurg. Psychiatry.

[5] Lindenbach D., Bishop C. (2013). Critical involvement of the motor cortex in the pathophysiology and treatment of Parkinson’s disease. Neurosci. Biobehav. Rev..

[6] Nagao S., Yokota O., Nanba R., Takata H., Haraguchi T., Ishizu H., Ikeda C., Takeda N., Oshima E., Sakane K. (2012). Progressive supranuclear palsy presenting as primary lateral sclerosis but lacking parkinsonism, gaze palsy, aphasia, or dementia. J. Neurol. Sci..

[7] Rossi S., Hallett M., Rossini P.M., Pascual-Leone A. (2009). Safety, ethical considerations, and application guidelines for the use of transcranial magnetic stimulation in clinical practice and research. Clin. Neurophysiol..

[8] Rossini P.M., Burke D., Chen R., Cohen L.G., Daskalakis Z., Di Iorio R., Di Lazzaro V., Ferreri F., Fitzgerald P.B., George M.S. (2015). Non-invasive electrical and magnetic stimulation of the brain, spinal cord, roots and peripheral nerves: Basic principles and procedures for routine clinical and research application. An updated report from an I.F.C.N. Committee. Clin. Neurophysiol. Off. J. Int. Fed. Clin. Neurophysiol..

[9] Cantone M., Lanza G., Le Pira A., Barone R., Pennisi G., Bella R., Pennisi M., Fiumara A. (2019). Adjunct Diagnostic Value of Transcranial Magnetic Stimulation in Mucopolysaccharidosis-Related Cervical Myelopathy: A Pilot Study. Brain Sci..

[10] Cantone M., Lanza G., Vinciguerra L., Puglisi V., Ricceri R., Fisicaro F., Vagli C., Bella R., Ferri R., Pennisi G. (2019). Age, Height, and Sex on Motor Evoked Potentials: Translational Data from a Large Italian Cohort in a Clinical Environment. Front. Hum. Neurosci..

[11] Lanza G., Cantone M., Puglisi V., Vinciguerra L., Fisicaro F., Vagli C., Bella R., Pennisi G., Di Lazzaro V., Pennisi M. (2019). “Mute” plantar response: Does the cortico-spinal tract “speak”?. Brain Stimul..

[12] Lanza G., Bella R., Giuffrida S., Cantone M., Pennisi G., Spampinato C., Giordano D., Malaguarnera G., Raggi A., Pennisi M. (2013). Preserved transcallosal inhibition to transcranial magnetic stimulation in nondemented elderly patients with leukoaraiosis. BioMed Res. Int..

[13] Bella R., Cantone M., Lanza G., Ferri R., Vinciguerra L., Puglisi V., Pennisi M., Ricceri R., Di Lazzaro V., Pennisi G. (2016). Cholinergic circuitry functioning in patients with vascular cognitive impairment—No dementia. Brain Stimul..

[14] Lanza G., Bella R., Cantone M., Pennisi G., Ferri R., Pennisi M. (2018). Cognitive Impairment and Celiac Disease: Is Transcranial Magnetic Stimulation a Trait d’Union between Gut and Brain?. Int. J. Mol. Sci..

[15] Lanza G., Lanuzza B., Aricò D., Cantone M., Cosentino F.I.I., Bella R., Pennisi G., Ferri R., Pennisi M. (2018). Impaired short-term plasticity in restless legs syndrome: A pilot rTMS study. Sleep Med..

[16] Fisicaro F., Lanza G., Grasso A.A., Pennisi G., Bella R., Paulus W., Pennisi M. (2019). Repetitive transcranial magnetic stimulation in stroke rehabilitation: Review of the current evidence and pitfalls. Ther. Adv. Neurol. Disord..

[17] Fisicaro F., Lanza G., Bella R., Pennisi M. (2020). “Self-Neuroenhancement”: The Last Frontier of Noninvasive Brain Stimulation?. J. Clin. Neurol..

[18] Kobayashi M., Pascual-Leone A. (2003). Transcranial magnetic stimulation in neurology. Lancet Neurol..

[19] Cantone M., Di Pino G., Capone F., Piombo M., Chiarello D., Cheeran B., Pennisi G., Di Lazzaro V. (2014). The contribution of transcranial magnetic stimulation in the diagnosis and in the management of dementia. Clin. Neurophysiol..

[20] Reis J., Swayne O.B., Vandermeeren Y., Camus M., Dimyan M.A., Harris-Love M., Perez M.A., Ragert P., Rothwell J.C., Cohen L.G. (2008). Contribution of transcranial magnetic stimulation to the understanding of cortical mechanisms involved in motor control. J. Physiol..

[21] Bella R., Ferri R., Lanza G., Cantone M., Pennisi M., Puglisi V., Vinciguerra L., Spampinato C., Mazza T., Malaguarnera G. (2013). TMS follow-up study in patients with vascular cognitive impairment-no dementia. Neurosci. Lett..

[22] Bella R., Lanza G., Cantone M., Giuffrida S., Puglisi V., Vinciguerra L., Pennisi M., Ricceri R., D’Agate C.C., Malaguarnera G. (2015). Effect of a Gluten-Free Diet on Cortical Excitability in Adults with Celiac Disease. PLoS ONE.

[23] Pennisi M., Lanza G., Cantone M., Ricceri R., Spampinato C., Pennisi G., Di Lazzaro V., Bella R. (2016). Correlation between Motor Cortex Excitability Changes and Cognitive Impairment in Vascular Depression: Pathophysiological Insights from a Longitudinal TMS Study. Neural Plast..

[24] Pennisi M., Lanza G., Cantone M., Ricceri R., Ferri R., D’Agate C.C., Pennisi G., Di Lazzaro V., Bella R. (2017). Cortical involvement in celiac disease before and after long-term gluten-free diet: A Transcranial Magnetic Stimulation study. PLoS ONE.

[25] Pierantozzi M., Panella M., Palmieri M.G., Koch G., Giordano A., Marciani M.G., Bernardi G., Stanzione P., Stefani A. (2004). Different TMS patterns of intracortical inhibition in early onset Alzheimer dementia and frontotemporal dementia. Clin. Neurophysiol. Off. J. Int. Fed. Clin. Neurophysiol..

[26] Rossini P.M., Rossi S., Babiloni C., Polich J. (2007). Clinical neurophysiology of aging brain: From normal aging to neurodegeneration. Prog. Neurobiol..

[27] Paulus W., Classen J., Cohen L.G., Large C.H., Di Lazzaro V., Nitsche M., Pascual-Leone A., Rosenow F., Rothwell J.C., Ziemann U. (2008). State of the art: Pharmacologic effects on cortical excitability measures tested by transcranial magnetic stimulation. Brain Stimulat..

[28] Cantello R. (2002). Applications of transcranial magnetic stimulation in movement disorders. J. Clin. Neurophysiol. Off. Publ. Am. Electroencephalogr. Soc..

[29] Gaspar P., Duyckaerts C., Alvarez C., Javoy-Agid F., Berger B. (1991). Alterations of dopaminergic and noradrenergic innervations in motor cortex in Parkinson’s disease. Ann. Neurol..

[30] Moore R.Y., Whone A.L., Brooks D.J. (2008). Extrastriatal monoamine neuron function in Parkinson’s disease: An 18F-dopa PET study. Neurobiol. Dis..

[31] Nagano-Saito A., Kato T., Arahata Y., Washimi Y., Nakamura A., Abe Y., Yamada T., Iwai K., Hatano K., Kawasumi Y. (2004). Cognitive- and motor-related regions in Parkinson’s disease: FDOPA and FDG PET studies. NeuroImage.

[32] Underwood C.F., Parr-Brownlie L.C. (2021). Primary motor cortex in Parkinson’s disease: Functional changes and opportunities for neurostimulation. Neurobiol. Dis..

[33] Leon-Sarmiento F.E., Rizzo-Sierra C.V., Bayona E.A., Bayona-Prieto J., Doty R.L., Bara-Jimenez W. (2013). Novel mechanisms underlying inhibitory and facilitatory transcranial magnetic stimulation abnormalities in Parkinson’s disease. Arch. Med. Res..

[34] Ni Z., Bahl N., Gunraj C.A., Mazzella F., Chen R. (2013). Increased motor cortical facilitation and decreased inhibition in Parkinson disease. Neurology.

[35] Barbin L., Leux C., Sauleau P., Meyniel C., Nguyen J.-M., Pereon Y., Damier P. (2013). Non-homogeneous effect of levodopa on inhibitory circuits in Parkinson’s disease and dyskinesia. Parkinsonism Relat. Disord..

[36] MacKinnon C.D., Gilley E.A., Weis-McNulty A., Simuni T. (2005). Pathways mediating abnormal intracortical inhibition in Parkinson’s disease. Ann. Neurol..

[37] Morita Y., Osaki Y., Doi Y. (2008). Transcranial magnetic stimulation for differential diagnostics in patients with parkinsonism. Acta Neurol. Scand..

[38] Sailer A., Molnar G.F., Paradiso G., Gunraj C.A., Lang A.E., Chen R. (2003). Short and long latency afferent inhibition in Parkinson’s disease. Brain J. Neurol..

[39] Manganelli F., Vitale C., Santangelo G., Pisciotta C., Iodice R., Cozzolino A., Dubbioso R., Picillo M., Barone P., Santoro L. (2009). Functional involvement of central cholinergic circuits and visual hallucinations in Parkinson’s disease. Brain J. Neurol..

[40] Celebi O., Temuçin C.M., Elibol B., Saka E. (2012). Short latency afferent inhibition in Parkinson’s disease patients with dementia. Mov. Disord. Off. J. Mov. Disord. Soc..

[41] Nardone R., Florio I., Lochner P., Tezzon F. (2005). Cholinergic cortical circuits in Parkinson’s disease and in progressive supranuclear palsy: A transcranial magnetic stimulation study. Exp. Brain Res..

[42] Ridding M.C., Inzelberg R., Rothwell J.C. (1995). Changes in excitability of motor cortical circuitry in patients with Parkinson’s disease. Ann. Neurol..

[43] Cantello R., Gianelli M., Bettucci D., Civardi C., De Angelis M.S., Mutani R. (1991). Parkinson’s disease rigidity: Magnetic motor evoked potentials in a small hand muscle. Neurology.

[44] Valls-Solé J., Pascual-Leone A., Brasil-Neto J.P., Cammarota A., McShane L., Hallett M. (1994). Abnormal facilitation of the response to transcranial magnetic stimulation in patients with Parkinson’s disease. Neurology.

[45] Abbruzzese G., Tabaton M., Morena M., Dall’Agata D., Favale E. (1991). Motor and sensory evoked potentials in progressive supranuclear palsy. Mov. Disord. Off. J. Mov. Disord. Soc..

[46] Kühn A.A., Grosse P., Holtz K., Brown P., Meyer B.-U., Kupsch A. (2004). Patterns of abnormal motor cortex excitability in atypical parkinsonian syndromes. Clin. Neurophysiol..

[47] Conte A., Belvisi D., Bologna M., Ottaviani D., Fabbrini G., Colosimo C., Williams D.R., Berardelli A. (2012). Abnormal cortical synaptic plasticity in primary motor area in progressive supranuclear palsy. Cereb. Cortex.

[48] Wittstock M., Pohley I., Walter U., Grossmann A., Benecke R., Wolters A. (2013). Interhemispheric inhibition in different phenotypes of progressive supranuclear palsy. J. Neural Transm..

[49] Udupa K., Chen R. (2019). Motor cortical circuits in Parkinson disease and dystonia. Handb. Clin. Neurol..

[50] Brusa L., Ponzo V., Mastropasqua C., Picazio S., Bonnì S., Di Lorenzo F., Iani C., Stefani A., Stanzione P., Caltagirone C. (2014). Theta burst stimulation modulates cerebellar-cortical connectivity in patients with progressive supranuclear palsy. Brain Stimulat..

[51] Bologna M., Bertram K., Paparella G., Papi C., Belvisi D., Conte A., Suppa A., Williams D.R., Berardelli A. (2017). Reversal of long term potentiation-like plasticity in primary motor cortex in patients with progressive supranuclear palsy. Clin. Neurophysiol. Off. J. Int. Fed. Clin. Neurophysiol..

[52] Bologna M., Suppa A., Stasio F.D., Conte A., Fabbrini G., Berardelli A. (2017). Neurophysiological studies on atypical parkinsonian syndromes. Parkinsonism Relat. Disord..

[53] Cantello R., Tarletti R., Civardi C. (2002). Transcranial magnetic stimulation and Parkinson’s disease. Brain Res. Brain Res. Rev..

[54] Oldfield R.C. (1971). The assessment and analysis of handedness: The Edinburgh inventory. Neuropsychologia.

[55] Postuma R.B., Berg D., Stern M., Poewe W., Olanow C.W., Oertel W., Obeso J., Marek K., Litvan I., Lang A.E. (2015). MDS clinical diagnostic criteria for Parkinson’s disease. Mov. Disord. Off. J. Mov. Disord. Soc..

[56] Höglinger G.U., Respondek G., Stamelou M., Kurz C., Josephs K.A., Lang A.E., Mollenhauer B., Müller U., Nilsson C., Whitwell J.L. (2017). Clinical diagnosis of progressive supranuclear palsy: The movement disorder society criteria. Mov. Disord. Off. J. Mov. Disord. Soc..

[57] Cummings J.L., Henchcliffe C., Schaier S., Simuni T., Waxman A., Kemp P. (2011). The role of dopaminergic imaging in patients with symptoms of dopaminergic system neurodegeneration. Brain J. Neurol..

[58] Nigro S., Arabia G., Antonini A., Weis L., Marcante A., Tessitore A., Cirillo M., Tedeschi G., Zanigni S., Calandra-Buonaura G. (2017). Magnetic Resonance Parkinsonism Index: Diagnostic accuracy of a fully automated algorithm in comparison with the manual measurement in a large Italian multicentre study in patients with progressive supranuclear palsy. Eur. Radiol..

[59] Fahn S. (1987). Unified Parkinson’s Disease Rating Scale. Recent Development in Parkinson’s Disease.

[60] Hoehn M.M., Yahr M.D. (1967). Parkinsonism: Onset, progression and mortality. Neurology.

[61] American Psychiatric Association (2013). Diagnostic and Statistical Manual of Mental Disorders.

[62] Folstein M.F., Folstein S.E., McHugh P.R. (1975). “Mini-mental state”. A practical method for grading the cognitive state of patients for the clinician. J. Psychiatr. Res..

[63] Dubois B., Slachevsky A., Litvan I., Pillon B. (2000). The FAB: A Frontal Assessment Battery at bedside. Neurology.

[64] Hamilton M. (1960). A rating scale for depression. J. Neurol. Neurosurg. Psychiatry.

[65] Ziemann U. (2013). Pharmaco-transcranial magnetic stimulation studies of motor excitability. Handb. Clin. Neurol..

[66] Ziemann U., Reis J., Schwenkreis P., Rosanova M., Strafella A., Badawy R., Müller-Dahlhaus F. (2015). TMS and drugs revisited 2014. Clin. Neurophysiol. Off. J. Int. Fed. Clin. Neurophysiol..

[67] Faro A., Giordano D., Kavasidis I., Pino C., Spampinato C., Cantone M.G., Lanza G., Pennisi M., Bamidis P.D., Pallikarakis N. (2010). An Interactive Tool for Customizing Clinical Transacranial Magnetic Stimulation (TMS) Experiments. IFMBE Proceedings, Proceedings of the XII Mediterranean Conference on Medical and Biological Engineering and Computing 2010, Chalkidiki, Greece, 27–30 May 2010.

[68] Cohen J. (1988). Statistical Power Analysis for the Behavioral Sciences.

[69] Lanza G., Kosac A., Trajkovic G., Whittaker R.G. (2017). Nerve Conduction Studies as a Measure of Disease Progression: Objectivity or Illusion?. J. Neuromuscul. Dis..

[70] Halliday G.M., Macdonald V., Henderson J.M. (2005). A comparison of degeneration in motor thalamus and cortex between progressive supranuclear palsy and Parkinson’s disease. Brain J. Neurol..

[71] Priori A., Berardelli A., Inghilleri M., Accornero N., Manfredi M. (1994). Motor cortical inhibition and the dopaminergic system. Pharmacological changes in the silent period after transcranial brain stimulation in normal subjects, patients with Parkinson’s disease and drug-induced parkinsonism. Brain J. Neurol..

[72] Sasaki K., Shimura H., Itaya M., Tanaka R., Mori H., Mizuno Y., Kosik K.S., Tanaka S., Hattori N. (2009). Excitatory amino acid transporter 2 associates with phosphorylated tau and is localized in neurofibrillary tangles of tauopathic brains. FEBS Lett..

[73] Fan J., Vasuta O.C., Zhang L.Y.J., Wang L., George A., Raymond L.A. (2010). N-Methyl-d-aspartate receptor subunit- and neuronal-type dependence of excitotoxic signaling through post-synaptic density 95. J. Neurochem..

[74] Nutini M., Frazzini V., Marini C., Spalloni A., Sensi S.L., Longone P. (2011). Zinc pre-treatment enhances NMDAR-mediated excitotoxicity in cultured cortical neurons from SOD1(G93A) mouse, a model of amyotrophic lateral sclerosis. Neuropharmacology.

[75] Yahagi S., Kasai T. (1999). Motor evoked potentials induced by motor imagery reveal a functional asymmetry of cortical motor control in left- and right-handed human subjects. Neurosci. Lett..

[76] De Gennaro L., Cristiani R., Bertini M., Curcio G., Ferrara M., Fratello F., Romei V., Rossini P.M. (2004). Handedness is mainly associated with an asymmetry of corticospinal excitability and not of transcallosal inhibition. Clin. Neurophysiol..

[77] Kertesz A., Geschwind N. (1971). Patterns of pyramidal decussation and their relationship to handedness. Arch. Neurol..

[78] Nathan P.W., Smith M.C., Deacon P. (1990). The corticospinal tracts in man. Course and location of fibres at different segmental levels. Brain J. Neurol..

[79] Wassermann E.M., McShane L.M., Hallett M., Cohen L.G. (1992). Noninvasive mapping of muscle representations in human motor cortex. Electroencephalogr. Clin. Neurophysiol..

[80] Macdonell R.A., Shapiro B.E., Chiappa K.H., Helmers S.L., Cros D., Day B.J., Shahani B.T. (1991). Hemispheric threshold differences for motor evoked potentials produced by magnetic coil stimulation. Neurology.

[81] Triggs W.J., Calvanio R., Macdonell R.A., Cros D., Chiappa K.H. (1994). Physiological motor asymmetry in human handedness: Evidence from transcranial magnetic stimulation. Brain Res..

[82] Davidson T., Tremblay F. (2013). Hemispheric differences in corticospinal excitability and in transcallosal inhibition in relation to degree of handedness. PLoS ONE.

[83] Ferron L., Tremblay F. (2017). (Lack of) Corticospinal facilitation in association with hand laterality judgments. Exp. Brain Res..

[84] Shibuya K., Park S.B., Howells J., Huynh W., Noto Y.-I., Shahrizaila N., Matamala J.M., Vucic S., Kiernan M.C. (2017). Laterality of motor cortical function measured by transcranial magnetic stimulation threshold tracking. Muscle Nerve.

[85] Souza V.H., Baffa O., Garcia M.A.C. (2018). Lateralized asymmetries in distribution of muscular evoked responses: An evidence of specialized motor control over an intrinsic hand muscle. Brain Res..

[86] Christakos C.N., Erimaki S., Anagnostou E., Anastasopoulos D. (2009). Tremor-related motor unit firing in Parkinson’s disease: Implications for tremor genesis. J. Physiol..

[87] de Carvalho M. (2012). Testing upper motor neuron function in amyotrophic lateral sclerosis: The most difficult task of neurophysiology. Brain J. Neurol..

[88] Hu X., Suresh A.K., Rymer W.Z., Suresh N.L. (2015). Assessing altered motor unit recruitment patterns in paretic muscles of stroke survivors using surface electromyography. J. Neural Eng..

[89] Issa N.P., Frank S., Roos R.P., Soliven B., Towle V.L., Rezania K. (2017). Intermuscular coherence in amyotrophic lateral sclerosis: A preliminary assessment. Muscle Nerve.

[90] Mitchell M., Martin B.J., Adamo D.E. (2017). Upper Limb Asymmetry in the Sense of Effort Is Dependent on Force Level. Front. Psychol..

[91] Sulena, Gupta D., Sharma A.K., Kumar N. (2017). Clinical Profile of Cognitive Decline in Patients with Parkinson’s Disease, Progressive Supranuclear Palsy, and Multiple System Atrophy. J. Neurosci. Rural Pract..

[92] Guerra A., Petrichella S., Vollero L., Ponzo D., Pasqualetti P., Määttä S., Mervaala E., Könönen M., Bressi F., Iannello G. (2015). Neurophysiological features of motor cortex excitability and plasticity in Subcortical Ischemic Vascular Dementia: A TMS mapping study. Clin. Neurophysiol..

[93] Pennisi G., Bella R., Lanza G. (2015). Motor cortex plasticity in subcortical ischemic vascular dementia: What can TMS say?. Clin. Neurophysiol. Off. J. Int. Fed. Clin. Neurophysiol..

[94] List J., Duning T., Kürten J., Deppe M., Wilbers E., Flöel A. (2013). Cortical plasticity is preserved in nondemented older individuals with severe ischemic small vessel disease. Hum. Brain Mapp..

[95] Lanza G., Bramanti P., Cantone M., Pennisi M., Pennisi G., Bella R. (2017). Vascular Cognitive Impairment through the Looking Glass of Transcranial Magnetic Stimulation. Behav. Neurol..

[96] Marchese R., Trompetto C., Buccolieri A., Abbruzzese G. (2000). Abnormalities of motor cortical excitability are not correlated with clinical features in atypical parkinsonism. Mov. Disord..

